# Supplementary material for: The Amino Acid Permease MoGap1 Regulates TOR Activity and Autophagy in Magnaporthe oryzae
Source: Int J Mol Sci. 2022 Nov 7;23(21):13663. doi: 10.3390/ijms232113663 (PMC9655246; doi:10.3390/ijms232113663)
Supplement: Supplementary file 1 [file ijms-23-13663-s001.zip › ijms-1988323-supplementary.pdf]

The Amino Acid Permease MoGap1 Regulates TOR Activity and Autophagy in *Magnaporthe oryzae*

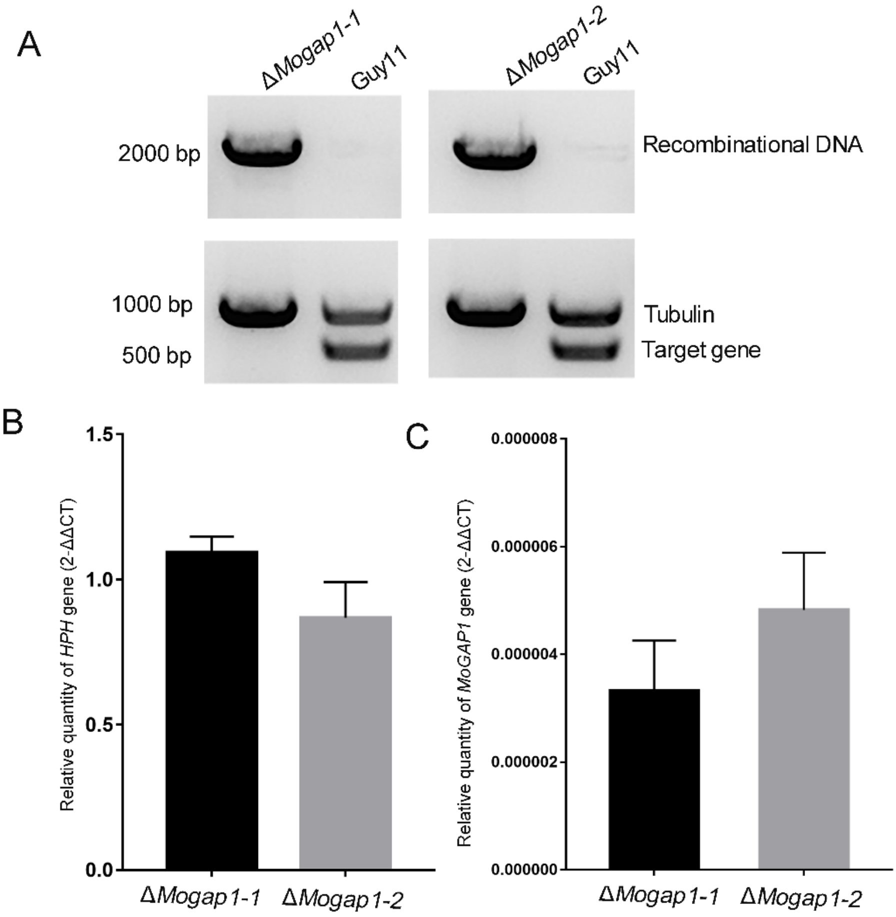

Figure S1. The knock-out strategy of *MoGAP1* in *M. oryzae*. **(A)** The recombinational DNA could be detected in the 2000 bp position in Δ*Mogap1* mutant while not in the wild-type Guy11 strain. The target gene can be detected in the wild-type Guy11 strain but not in *MoGAP1* deletion mutant. **(B)** The relative expression of *HPH* gene in *MoGAP1* deletion mutant using qPCR. **(C)** The relative expression of *MoGAP1* gene in *MoGAP1* deletion mutant using qPCR.

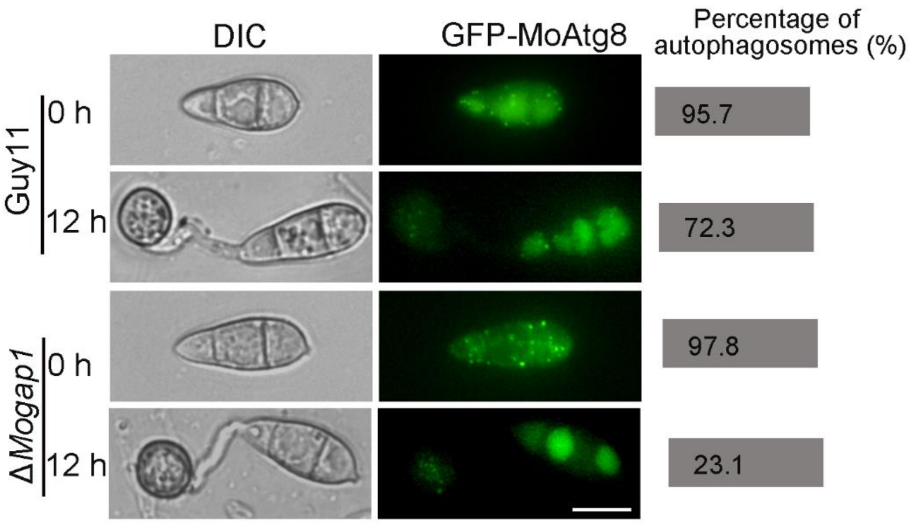

Figure S2. The autophagy flux was observed in conidia. The conidia of the wild-type Guy11 and Δ Mogap1 mutant were collected and dropped on glass slide for 0 h and 12 h. Bar = 10 μm.
